# A rare case of congenital pulmonary airway malformation in a 14-year-old male presenting with spontaneous pneumothorax

**DOI:** 10.1016/j.amsu.2021.102692

**Published:** 2021-08-05

**Authors:** Bashar Aljarad, Issam Alkhayer, Ahmad Alturk, Safaa Qatleesh, Albaraa Bara

**Affiliations:** a Damascus University, Damascus, Syrian Arab Republic; b Department of Thoracic Surgery, Al- Mouwasat Hospital, Damascus, Syrian Arab Republic; c Department of Pathology, Al- Mouwasat Hospital, Damascus, Syrian Arab Republic

**Keywords:** Congenital cystic adenomatoid malformation, CPAM, Spontaneous pneumothorax, Adult, Case report

## Abstract

**Introduction:**

and importance: Congenital pulmonary airway malformation (CPAM), formerly known as congenital cystic adenomatoid malformation (CCAM), is a rare developmental dysplatic lesion of the fetal tracheobronchial tree. It accounts for approximately 25 % of all congenital lung malformations. It is usually unilateral and involves one lobe with no significant gender or racial predilection. The vast majority of reported CPAM cases were discovered prenatally or within the first 2 years of life; however, it is rarely found in older children and adults.

**Case presentation:**

The purpose of this paper is to present a case of a 14-year-old male with a chest tube inserted 5 days before, as a management to left-sided spontaneous pneumothorax. His vital signs and laboratory tests were all within normal. Chest X-ray showed irregular opacity in the left lung field.

**Clinical discussion:**

CT revealed multiple cystic-like lesions in the upper lobe of the left lung. The clinical impression was pointing towards a congenital lung lesion. The patient then underwent surgery. There were several pleural adhesions. The adhesions were released, and the upper left lobe was resected. Histopathological findings were compatible with type II CPAM. Four days postoperatively, chest X-ray was within the normal.

**Conclusion:**

We report this case to highlight the importance of considering CPAM and other congenital malformations as a differential diagnosis in the adult population, especially in patients with sudden onset of pulmonary symptoms along with multiple cystic-like lesions on CT, as well as to draw attention towards spontaneous pneumothorax as a possible first presentation for CPAM.

## Highlights

• CPAM is a rare developmental dysplatic lesion of the fetal tracheobronchial tree.
• CPAM is usually unilateral and involves one lobe.
• Consider congenital lung malformations in patients with sudden pulmonary symptoms.
• CPAM could rarely found in older children and adults.
• Spontaneous pneumothorax could be the first presentation in patient with CPAM.

## 1 Introduction

Congenital cystic adenomatoid malformation (CCAM) is a rare developmental dysplatic lesion of the fetal tracheobronchial tree. It was first described in 1897 by Stoerk et al. [1], when they reported a cystic formation in the lung of a newborn, but it was not until 1949 that CCAM was defined as a distinct disease by Ch'in and Tang [2]. In 1977, Stocker et al. classified CCAM into 3 subtypes [3]; then, Stocker reclassified it in 2002 into 5 subtypes based on morphological and histopathological features and renamed it to ‘congenital pulmonary airway malformation’ (CPAM) [4]. Although rare, it accounts for approximately 25 % of all congenital lung malformations [5]. It is usually unilateral and involves one lobe with no significant gender or racial predilection [6]. The vast majority of reported cases of CPAM were discovered prenatally or within the first 2 years of life; however, it is rarely found in older children and adults [7]. In this paper, we report a case of CPAM in a 14-year-old patient that has been managed surgically. What makes our case significant is the age of the patient, as almost all CPAM cases are diagnosed prenatally or during the neonatal period. This work has been reported in line with the SCARE criteria [8].

## 2 Case presentaion

A 14-year-old male came to our hospital with a chest tube inserted 5 days before, when he was diagnosed with spontaneous pneumothorax after complaining of sudden left-sided chest pain and dyspnea. His medical history included recurrent periods of dry cough in the last 2 years; otherwise, his surgical, familial, and social history were negative.

On physical examination, the patient was conscious, aware, responsive to stimuli, and in a good general condition. His blood pressure, heart rate, temperature, and O2 saturation were stable and within normal ranges. On chest auscultation, breathing sounds were diminished over the upper half of the left lung. Laboratory tests including C-reactive protein (CRP), erythrocyte sedimentation rate (ESR), complete blood count (CBC), prothrombin time (PT), and international normalized ratio (INR) were within normal ranges. Flexible bronchoscopy was normal, GeneXpert test for tuberculosis (TB) was negative.

**Fig. 1:**
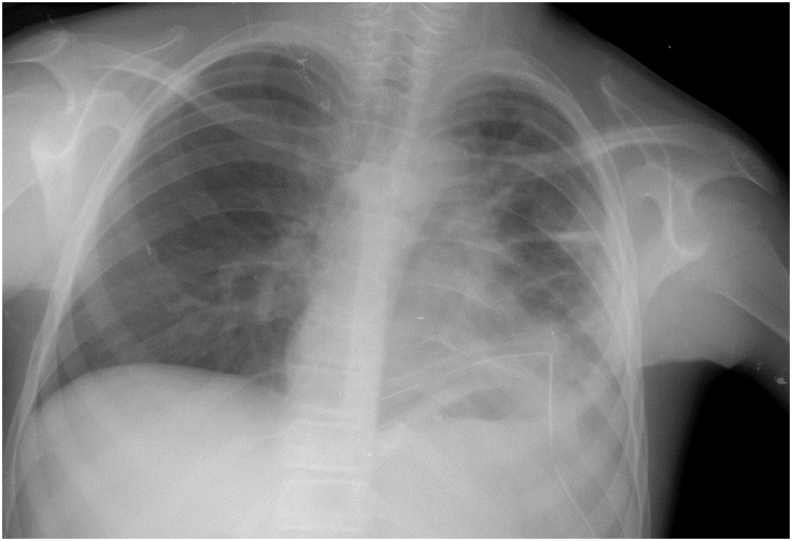
Patient's chest X-ray showing irregular opacity in the left lung field.

**Fig. 2:**
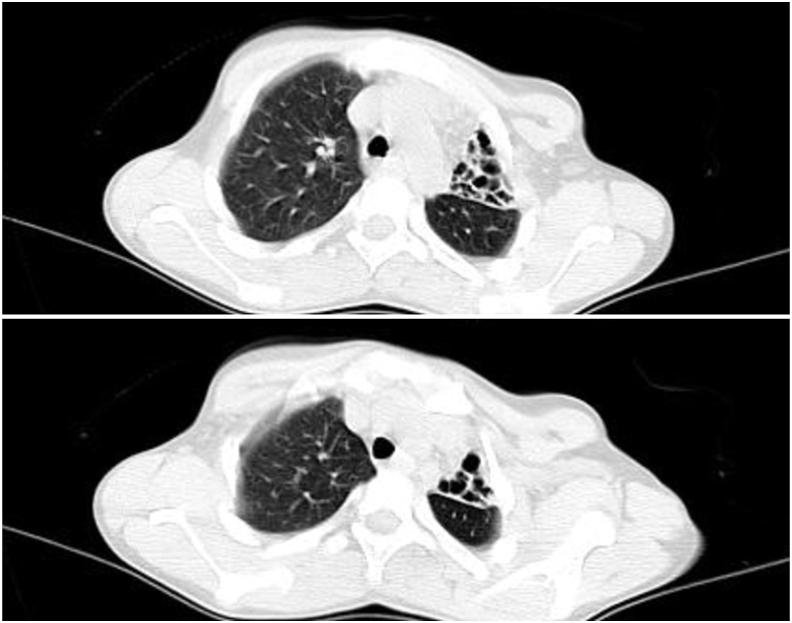
Patient's CT scan: multiple cystic-like lesions in the upper lobe of the left lung.

Chest X-ray showed irregular opacity in the left lung field (Fig. 1), which was revealed later on computed tomography (CT) as multiple cystic-like lesions in the upper lobe of the left lung (Fig. 2).

**Fig. 3:**
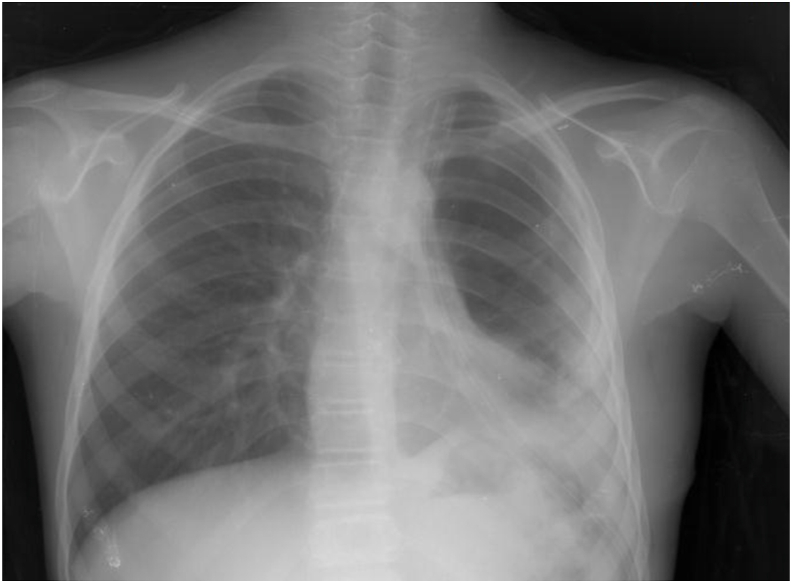
Normal chest X-ray four days after the surgery.

The patient then underwent surgery; we did a left posterolateral thoracotomy through the fifth intercostal space. There were several pleural adhesions, with the ones between the upper lobe and the mediastinum being severe. The adhesions were released, and the upper left lobe was resected. Routine lab tests were done post surgery, including coagulation studies (PT, PTT, INR), CBC, liver enzymes, and cardiac enzymes. All tests came out normal, and the follow-up chest X-ray four days postoperatively was also normal (Fig. 3). Overall, the patient had an uneventful recovery and had no findings worth mentioning.

Macroscopically, the patient had a 2.5 × 1.5 cm cyst located underneath the bronchus with multiple cystic lesions with no specific distribution.

**Fig. 4:**
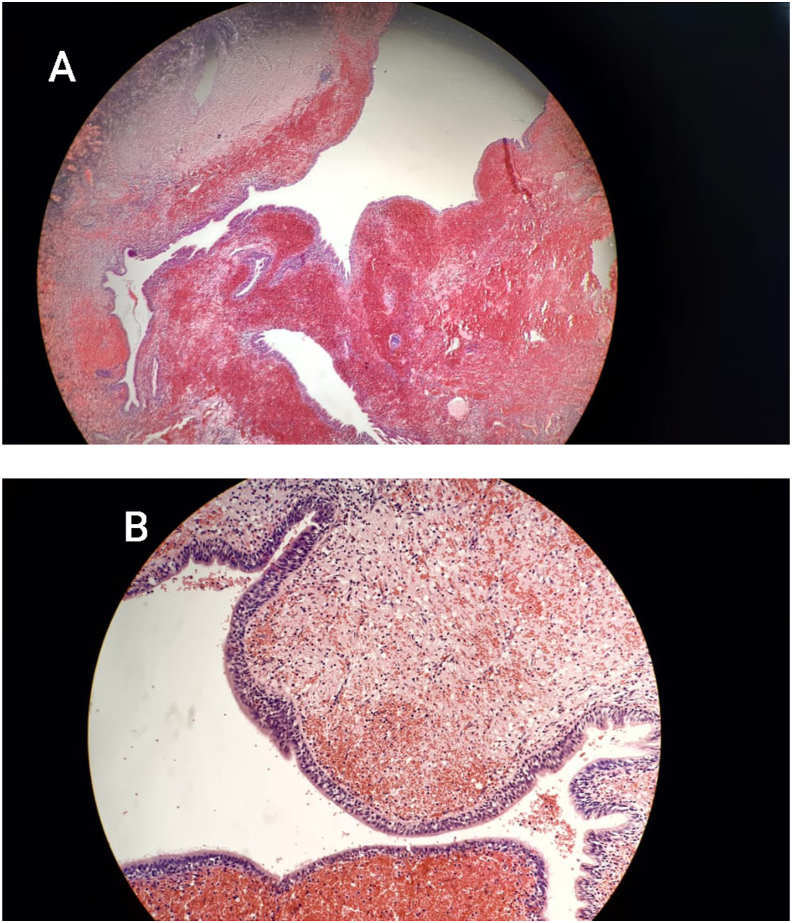
A: Variously sized Microcystic air spaces embedded, normal alveoli with congestion (Low power). B: Microcystic air spaces lined by respiratory epithelium (High power).

Histopathology revealed multiple variously sized cysts, one of them measures 2.5 cm in the greatest dimention, lined by cuboidal-to-ciliated pseudostratified columnar epithelium, some of them resemble bronchioles. The cysts were separated by normal alveoli with severe congestion and interstitial hemorrhage. No granulomas or destruction of parenchyma were noted (Fig. 4). There were no signs of malignancy. These findings were compatible with type II CPAM.

## 3 Discussion

Congenital cystic adenomatoid malformation (CCAM) is a congenital formation of cystic lesions caused by adenomatous hyperplasia in the respiratory tract epithelium [9].In 1977, CCAM was categorized by Stocker into three groups according to histopathological and morphological specifications [10].The vast majority of CCAM patients have type I, representing 70 % of all CCAM patients. Type I CCAM lesions measure >3 cm; it could be a single cyst or multi-cystic lesions with pseudostratified ciliated columnar epithelium, which includes mucous cells that could be under the potential risk of malignant transformation to adenocarcinoma. Type II CCAM lesions are uniform-sized multi-cystic formations measuring <2 cm located in the distal part of the respiratory tract, with cuboidal to columnar epithelium. While type III CCAM bulky lesion occupies an entire lobe or even the whole lung, the cysts scattered inside it usually measure less than 0.3–0.5 cm [11].

In 2002, Stocker gave CCAM a new name as ‘congenital pulmonary airway malformation’ (CPAM) and expanded his classification into 5 types to include: type 0, which consists of solid bilateral lesions; and type IV that appear as a huge marginal cystic lesion [10]. Nevertheless, this new categorization still did not spread widely.

CPAM can manifest in various ways, with respiratory distress being the most common clinical presentation in neonates, regardless of which type of CPAM the neonate has. In about 50 % of affected newborns, CPAM can remain clinically silent until later in life; then, it could manifest as hemoptysis, dyspnea, chest pain, and cough, or it could be discovered coincidentally during imaging for a different reason or while investigating the etiology behind recurrent respiratory infections; spontaneous pneumothorax could also occur with CPAM [9,12]. In our case, the patient had cough, pneumothorax, dyspnea, and chest pain.

Sometimes it could be diffecult to discriminate CPAM from other abnormalities which represent the differential diagnosis of CPAM like: bronchogenic cysts, bronchiectasis, bronchial atresia, and pulmonary sequestration, especially when the radiological investigations mistakenly reveal CPAM as another disease that tends to spread widely throughout adults society; mainly if it appears as a single, localized solid or cystic-like lesion. Moreover, using ultrasound is considered to be helpful sometimes to detect CPAM in newborns. Another worthy investigation is taking a biopsy from the radiologically enhanced lesion, which has been proved to be beneficial in raising the diagnosis rate up to 98.3 % [9,10].

Surgical resection is considered the treatment of choice for CPAM to confirm the diagnosis and avoid the high potential of pulmonary infection and turning into malignancy [12].

To the best of our knowledge, CPAM is rarely found in older children and adults, which makes our case of CPAM in a 14-year-old patient an uncommon manifestation.

## 4 Conclusion

We report this case to highlight the importance of considering CPAM and other congenital malformations as a differential diagnosis in the adult population, especially in patients with sudden onset of pulmonary symptoms along with multiple cystic-like lesions on CT, as well as to draw attention towards spontaneous pneumothorax as a possible first presentation for CPAM.

## Sources of funding

None.

## Research registration

None.

## Ethical approval

No ethical approval was needed.

## Consent

Written informed consent was obtained from patient's mother for publication of this case report and accompanying images. A copy of the written consent is available for review by the Editor-in-Chief of this journal on request.

## Author contribution

BA: reviewed the literature, wrote the case presentation, and the discussion. IA: led the surgical team, checked the spelling and grammar, and critically revised the manuscript. AA: reviewed the literature, and wrote the introduction. SQ: did the histopathological study, and designed the figures. AB: reviewed the literature, wrote the abstract and the conclusion. Guarantor: AA.

## Sources of funding

There was no funding.

## Registration of research studies

N/A.

## Guarantor

Dr. Ahmad Alturk.

## Declaration of competing interest

All of the authors declared that they have no conflict of interest.

Provenance and peer review: Not commissioned, externally peer-reviewed.
